# Sediment Carbon Accumulation in Southern Latitude Saltmarsh Communities of Tasmania, Australia

**DOI:** 10.3390/biology7020027

**Published:** 2018-05-02

**Authors:** Joanna C. Ellison, Kim M. Beasy

**Affiliations:** 1 Discipline of Geography and Spatial Sciences, School of Technology, Environments and Design, University of Tasmania, Launceston, Tasmania 7250, Australia; 2 School of Education, University of Tasmania, Launceston, Tasmania 7250, Australia; Kim.Beasy@utas.edu.au

**Keywords:** saltmarsh, sediment carbon, wetland, accumulation rates, *Spartina*, pollen analysis

## Abstract

Carbon sequestration values of wetlands are greatest in their sediments. Northern hemisphere research dominates the earlier saltmarsh carbon sequestration literature, recently augmented by analyses across mainland Australia where species assemblages, catchment histories and environmental settings differ. No previous assessment has been made for Tasmania. Carbon stores and accumulation rates in saltmarsh sediments of the Rubicon estuary, Tasmania, were investigated. Carbon was determined from sediment cores by Elemental Analyser, combined with analysis of organic content and bulk density. Carbon accumulation was determined using short-term and long-term sediment accretion indicators. Results showed carbon densities to be lower than global averages, with variation found between carbon stores of native and introduced species zones. Cores from introduced *Spartina anglica* indicated a trend of higher sediment carbon percentages relative to cores from native saltmarsh *Juncus kraussii* and *Sarcocornia quinqueflora*, and in finer grain sizes. Sediment carbon stock of 30 cm depths was 49.5 Mg C ha^−1^ for native saltmarsh and 55.5 Mg C ha^−1^ for *Spartina*. Carbon percentages were low owing to high catchment inorganic sediment yields, however carbon accumulation rates were similar to global averages, particularly under *Spartina*. Covering 85% of saltmarsh area in the estuary, *Spartina* contributes the majority to carbon stores, potentially indicating a previously unrecognized value for this invasive species in Australia.

## 1. Introduction

Coastal wetlands are globally valued for their protection of land through reducing wind and wave energy, as well as being integral to marine communities through the provision of organic matter [1,2]. This century, coastal wetlands have been shown to be important carbon sinks [3], being amongst the highest carbon storing ecosystems globally [4,5] contributing a disproportionately high sink for enhanced greenhouse gas emissions relative to the wetland global area [3,6]. The largest component of organic carbon found in coastal wetland systems is in the sediment profile [7,8,9] where organic carbon accumulates from the decomposition of leaves, stems and root matter of wetland vegetation during sediment accretion [10,11]. The ability of coastal wetlands to store carbon has provided an additional conservation value in a period when the vulnerability of these unique ecosystems is increasing due to degradation, erosion, reclamation, and accelerated sea level rise [3,7,9,12,13,14].

Saltmarshes provide a long-term carbon sink owing to increasing sediment volume over long timescales, with carbon sequestered from roots, rhizomes, leaves and stems of marsh grasses, in the water-saturated conditions of the sediment profile [15]. Of all coastal wetlands, the most significant data gaps remain for sediment carbon evaluation in tidal saltmarshes, even though they are likely to hold the highest long-term rate of carbon accumulation in sediment [16]. The amount of carbon found in sediment is variable based on local site conditions, and species [7,10,12]. Further research is required to improve global assessments of tidal saltmarsh carbon stocks, particularly in the southern hemisphere where very little data is available [7,12].

Analysis of saltmarsh data from sites between 28.4 and 55.5° N found an average carbon density in saltmarshes to be 0.039 ± 0.003 g cm^−3^ [7]. Belowground stocks of Southeast mainland Australia saltmarshes 29–37° S showed little variation with latitude [5], geomorphic settings rather being a control on carbon sequestration variation through influences on sedimentary factors. Fluvial settings showed more than two times higher carbon stocks relative to marine settings, attributed to finer sediment grain sizes [5]. Assessment of carbon stocks across mainland Australia also found that landward fluvial tidal marsh sites showed twice the amount of organic carbon relative to seaward, marine sites [17].

Limited research has however been published on saltmarsh carbon storage from the Southern hemisphere, with early data showing only one study reporting research further south than 32° in latitude [7,12,16]. In the last five years, understanding of variation in carbon stocks in NSW and Queensland has increased [5,18,19,20], as well as in Victoria [5,21], and data from all mainland Australian states and territories were analysed in the last 12 months to show an average of 77.9 Mg organic carbon (OC) per hectare (range 8.9–603.7 Mg C ha^−1^), in the top 30 cm of marsh sediment [17]. There is a need to collect data on organic carbon stocks in areas unrepresented in previous studies such as Tasmania [17], as there remains no assessment from this southern Australian Island State. There may be differences in southern hemisphere sites owing to different species assemblages, as well as geomorphic settings and catchment histories.

The aim of this study is to undertake an exploratory investigation into the sediment carbon store and carbon accretion rates of native and introduced estuarine saltmarsh species in Tasmania, the southernmost state of Australia at a latitude of 41°09′ S.

## 2. Materials and Methods

### 2.1. Study Site

**Figure 1:**
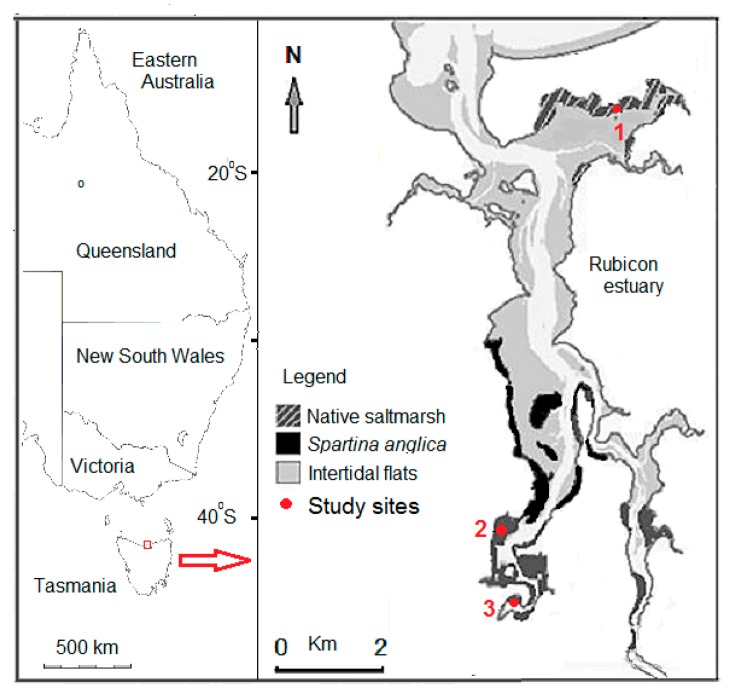
Location of the Rubicon estuary, Tasmania, showing intertidal zones and saltmarsh study sites.

Tasmania extends across a latitudinal range of 39°40′–43°20′ S, and has a total saltmarsh area of 47 km^2^ [22]. Tidal ranges are micro-tidal [23], but of greater range on the north coast, where saltmarsh extents are also greater relative to the south and west coasts. The Rubicon Estuary on the central north coast of Tasmania (41°09′ S, 146°33′ E; Figure 1) has a total catchment area of 262 km^2^, an overall low topographic relief and a tidal range of 2.1 m [24]. The mean monthly temperature range is 8.3–17 °C, and the mean precipitation is 773 mm a^−1^ with a winter maximum [25]. The climatic conditions within the greater Rubicon estuary area have been historically favorable to land use changes such as intensive agriculture, that have resulted in a high sediment supply [26,27].

Common saltmarsh species in Tasmania include *Sarcocornia quinqueflora*, which is restricted to Australasia. The plant has a high tolerance to saline soils and waterlogged conditions, and inhabits the lower saltmarsh zone [28]. *Juncus kraussii* is also a common saltmarsh species in Tasmania, and is not as salt tolerant as *S. quinqueflora* so inhabits the high saltmarsh zone [28]. *Juncus* species occur across Europe and America, but *J. kraussii* only occurs in the southern hemisphere in Australia, New Zealand and South Africa [29], where it inhabits the landward fringe of the saltmarsh. *Spartina anglica* originated in England, and was intentionally introduced last century in Europe, the USA, China, Australia, and to Tasmania in the 1940s [11]. *Spartina* can occupy both low and high saltmarsh zones, and is now considered to be a vigorous invasive species in Tasmania [30]. The Rubicon is the second largest infestation in Australia, after the Tamar estuary located to the east of the Rubicon.

Sites were chosen to undertake an exploratory investigation of these saltmarsh communities dominated by a particular species in the Rubicon Estuary. Site 1 was located in native saltmarsh (Figure 1), in an estuarine bay protected area managed as a *Spartina* free zone. Site 2 was located in the upper estuary, in dense pioneer cover of introduced *Spartina* vegetation with no native saltmarsh species present. Site 3 was located on the western margin of the Rubicon estuary in dense, well-established *Spartina* vegetation (Figure 1).

### 2.2. Sample Collection and Laboratory Analyses

Two cores were collected at site 1, core 1.1 from *J. kraussii* vegetation, and core 1.2 from *S. quinqueflora* vegetation, and one core was collected each from each of sites 2 and 3. A sidewall sampling peat corer was used to extract sediment cores, of 8.5 cm in diameter to a depth of approximately 0.6 m. Compaction was negligible owing to the sidewall sample design of the corer. The cores were placed in a split plastic pipes, taped and wrapped securely for transportation to the analytical laboratory. Cores were carried horizontally at all times to prevent vertical compaction. In the laboratory, cores were opened in a horizontal position and sediment was sampled at 5 cm increments.

Grain size was determined using the Bouyoucos method [31], and 2 depths were analysed per core, from 13–15 cm to 40–42 cm. Carbon density has been shown to decline with depth [5,12,15], and hence particle size distribution from samples above 15 cm and below 40 cm may indicate potential trends. Samples were oven dried at 60 °C for 48 h, then any roots removed through a 1.18 mm sieve. Samples were placed in a microid flask shaker with the dispersing agent 5% sodium hexametaphosphate for 10 min. Then samples were shaken in a volumetric flask with distilled water, and inverted to further disperse particles, and suspended solids measured with a hydrometer at 5 min and 2 h.

For determination of organic carbon content, pre-treatment of HCl was used for the removal of inorganic carbon from the samples prior to the carbon analysis [32]. Subsamples were immersed in 6N HCl to test for carbonates [32], and not to the entire sample, as HCl may dissolve part of the organic matter present within the sample, leading to an underestimation of the organic matter content [33]. Where effervescence was present, samples were immersed in 6N HCl for 10 min then rinsed 5 times with distilled water to remove remnant traces of HCl [32]. Samples not displaying effervescence were mixed and then divided by volume into 10 mL replicates for bulk density analysis [34]. After oven drying at 60 °C for 72 h until a constant dry weight was obtained [11], samples were passed through a 425 μm sieve to remove vegetation fragments before being ground into a fine powder using a mortar and pestle. Samples were then placed in a muffle furnace at 550 °C for 4 h [35]. Following air-drying, structural water accounts for only 2% of ignition loss in high clay content estuarine marsh soil [32].

To measure the concentration of carbon content of the sediment samples (%), replicate samples from each core were prepared as above [32] and analysed using a Thermo Finnigan EA 1112 Series Flash Elemental Analyser, after being weighed to a precision of 0.1 μg. The Elemental Analyser measured the amount of carbon, hydrogen and nitrogen in the material by the combustion of small amounts (1–2 mg) of the sediment sample in pure oxygen and in the presence of catalysts at high temperature (1000 °C). The combustion products were separated by passing them through a packed column and quantified using a thermal conductivity detector. Carbon density was determined from the bulk density results and the elemental analysis carbon percentage results.

Carbon contained in catchment or estuary sediment has previously been estimated using published saltmarsh area data [12]. Areas of native and *Spartina* saltmarshes within the Rubicon Estuary have been recently mapped at 1:500 or less using spatial analysis [36,37]. Carbon content was calculated for the top 30 cm of sediment to enable comparison with that elsewhere [12,16,17] using average results of carbon density (g cm^−3^) from cores from native marsh sites 1.1 and 1.2, average results for cores from *Spartina* sites 2 and 3, and areas of *Spartina* and native saltmarsh vegetation zones [36,37].

### 2.3. Vertical Accretion

Short-term sediment accretion can be measured using sediment accretion above an inserted marker horizon [12,16,18,19,38]. Three replicate feldspar plots (0.25 m by 0.25 m) were established on the marsh at each site. The locations of plots were based on the following criteria: representation of the species, accessibility and location in an area that was prograding and not in an erosion zone as shown by topographic profiles. In areas with dense plant cover, feldspar clay was shaken through vegetation until the marsh surface was adequately covered to a depth of 5 mm, feldspar plots were re-visited after 6 and 12 months [38], and the depth of accretion above the marker was measured to the nearest millimetre at three positions and averaged [12]. Samples were taken of the accumulated sediment above the feldspar horizon, and analysed to determine bulk density and to calculate a carbon density accumulation rate.

Pollen analysis was performed on the site 1.1 *J. kraussii* core in order to calculate a long-term accretion rate, as no visible sediment had accreted on the feldspar plot after 6 and 12 months. The depth in a core at which an introduced species first appears can be used to date that level [11,39], allowing a net accretion rate above this to be calculated, for the time period subsequently elapsed. This approach was used in the Tamar estuary to calculate net accretion rates over the last few decades [11], using historical information on the introduction of *Spartina*. The dates of introduction of *Spartina* to the Rubicon estuary are not recorded, however, *Pinus radiata* plantations were established in the Rubicon catchment from 1968, within 30 km of the study sites. *P. radiata* first flowers at the age of 7 [40], and is wind-pollinated. Therefore, it is likely that abundant pine pollen would appear in the record in 1976. Ten samples at 5 cm increments from the core were prepared using the standard pollen concentration technique [41]. A known amount of spores of the exotic Lycopodium was added to allow determination of pollen concentration. Pollen grains were identified, and counted at each sample level until a count of 200 g was reached. Net vertical accretion was calculated using the depth of first pine pollen appearance divided by the time elapsed since.

## 3. Results

### 3.1. Grain Size and Organic Matter

**Figure 2:**
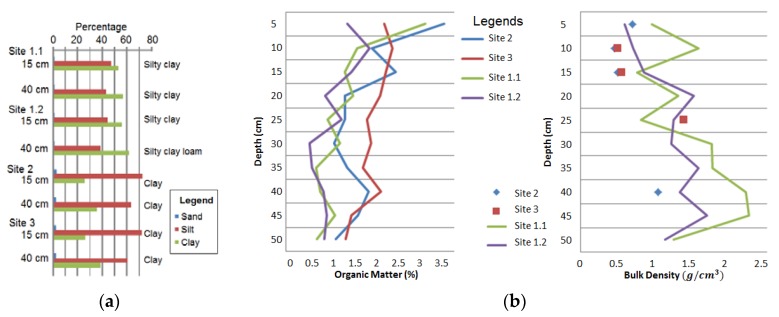
(**a**) Grain size results in percentages of sand, silt and clay, and texture classification; (**b**) Organic matter content and bulk density trends with depth from Rubicon estuary saltmarsh sites.

Grain size distributions of sediments in cores are shown for all sites in Figure 2a, with all sites showing decreasing clay and increasing silt with depth. The up-estuary *Spartina* sites 2 and 3 (Figure 1) showed all results to be clay, while both cores at site 1 showed coarser results of a silty clay combination.

Organic matter content decreased with depth at all sites (Figure 2b). Site 1.2 (*S. quinqueflora*) and Site 3 (*Spartina*) showed the most even distribution of organic matter decreasing by less than 1% with depth. Site 1.1 (*J. kraussii*) showed a higher surface organic content (3%), however, a lower mean organic content. Site 1.2 had the lowest mean organic content, while the loss-on ignition results from *Spartina* sites 2 and 3 were within 0.1% of each other (Table 1).

The bulk density results in each core showed an inverse relationship with organic matter content (Figure 2b), with values of 0.5–2.4 g cm^−3^ with depth. Bulk density was not calculated on all site 2 and 3 samples due to the pre-treatment of HCl, restricting analysis of a trend with depth. No effervescence was observed in native marsh cores from site 1, while minor effervescence was observed in six of the subsamples from *Spartina* cores, most of these from site 2 (Figure 1).

### 3.2. Sediment Carbon Results

**Figure 3:**
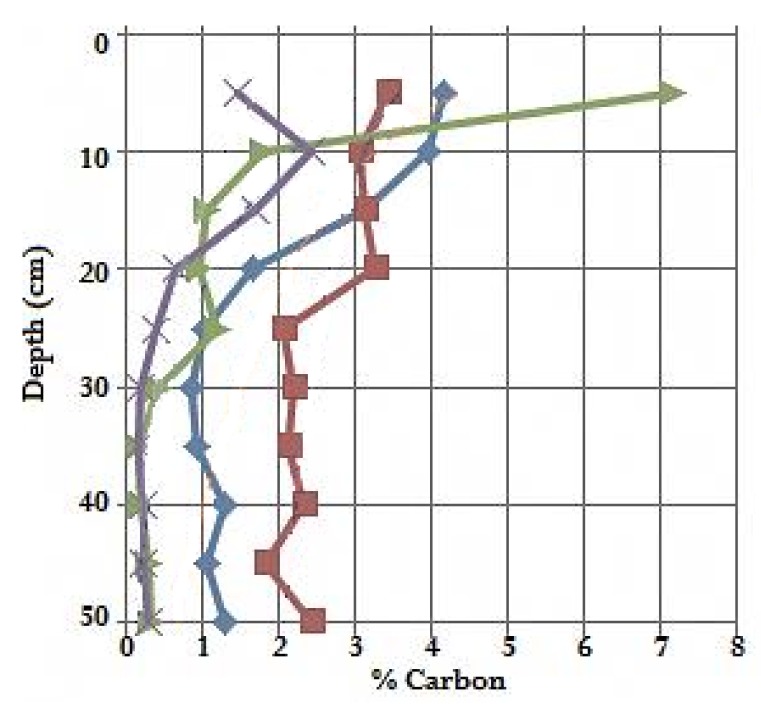
Relationship between percentage carbon with depth from each core.

Percentage carbon results with depth showed carbon levels of 0.5% to 7.0%, with decreasing carbon with depth at each site (Figure 3). Sites 2 and 3 both showed the most carbon stored throughout the sediment profile. However, site 1.1 (*J. kraussii*) showed the highest proportion of carbon in the surface sediment (0–5 cm) (7.16%).

**Figure 4:**
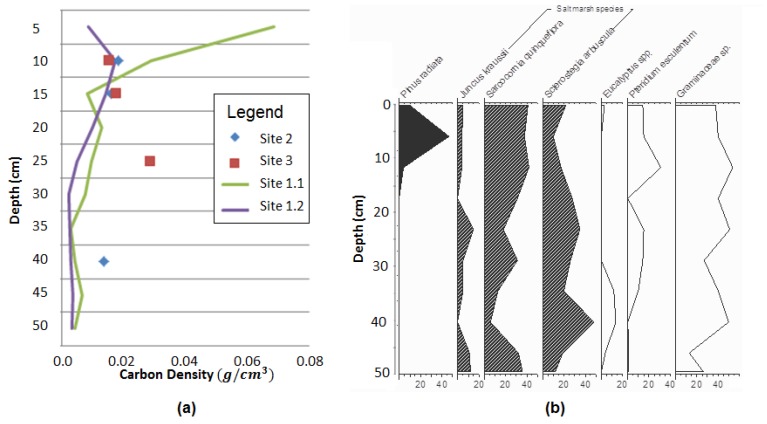
(**a**) Carbon density trends with depth in core samples from Rubicon estuary sites; (**b**) Pollen percentages with depth from the site 1.1 core.

Carbon density results with depth in each core (Figure 4a) showed that the density of carbon was greater in non-native *Spartina* marsh (sites 2 and 3) relative to the native marsh site 1.2 with *S. quinqueflora*, but less than the native high marsh site 1.1 with *J. kraussii* (Table 1). The density of carbon decreased with depth in cores from all sites. The core results from each site showed twice the carbon density in native high marsh *J. kraussii* sediment (site 1.1), relative to low marsh native *S. quinqueflora* at site 1.2. The non-native species core results were consistent in carbon density at both sites 2 and 3.

Vertical accretion and carbon accumulation results from core results from each site (Table 1) showed that the native low marsh species, *S. quinqueflora*, at site 1.2 accumulated at a slower rate than at the *Spartina* sites. Based on the 12 months accretion data, *Spartina* at site 3 showed net sediment accumulation rates at 150% the rate of *Spartina* at site 2. By contrast, the native high marsh species *J. kraussii* at site 1.1 showed 0 mm a^−1^ vertical accretion over the 1 year study duration using the feldspar method. Results from the alternative technique adopted of pollen analysis from the core at site 1.1 are shown in Figure 4b, with several saltmarsh species represented and pine pollen appearing at 20 cm depth, with 40% abundance at 10 cm depth. Pine plantations were established in the catchment in 1968 and were likely flowering by 1976, hence indicating that long term sediment accretion at site 1.1 has been at a rate of 0.42 mm a^−1^ (Table 1).

Spatial analysis data from the Rubicon estuary [36,37] for the area of this study (Figure 1) showed native saltmarsh to cover a total area of 39 hectares, with *Spartina* covering an area of 226 hectares in 2016 (Table 2). This area of *Spartina* is greater than previous estimates [42], and can be attributed to spread of *Spartina* in marginal sections such as site 2. An estuarine saltmarsh sediment carbon content was calculated based on the carbon density results (Table 2), showing variation according to native and introduced vegetation types and geomorphic settings.

## 4. Discussion

All cores from this study showed a fining upwards trend of increased clay (Figure 2a), and also finer grain sizes closer to fluvial sources rather than the estuary mouth (Figure 1 and Figure 2a), with all samples from the *Spartina* sites classified as clay sediments. Fluvially-dominated sites are found to have finer sediment sizes relative to marine-dominated sites [5]. Decreased grain size in sediments causes the sediment organic carbon to increase [5,43], owing to clay particles binding carbon molecules and reducing microbial breakdown [44].

The core results indicated organic matter in the top 30 cm of saltmarsh sediment to be higher in introduced *Spartina* sites relative to native saltmarsh in the Rubicon estuary (Figure 2b; Table 1), but lower relative to wetland sediment averages both globally and elsewhere in Australia [7]. The adjacent Tamar estuary located to the east of the Rubicon typically has 14–28% organic matter in *Spartina* sediment by comparison [11], determined using the same techniques as in this study. These low results from the Rubicon of 0.5–3.5% (Table 1) may be due to the high catchment sediment yield, with recent land use changes [26,27].

Carbon density results from the four cores analysed in this study from Rubicon estuary marshes (Figure 4a) indicated levels lower relative to the northern hemisphere, with analysis of sites between 28.4 and 55.5° N showing the global average carbon density for saltmarshes to be 0.039 g cm^−3^ [7], and also lower relative to NSW results from 38° S, of 0.03 g cm^−3^ [13]. Carbon density results were also at the lower range of results from locations of similar mean annual temperature [7], and lower than the fluvially-dominated saltmarshes of NSW (29–37° S) [5], which may be owing to the higher latitude of the Rubicon, and higher sediment accretion rates with mostly inorganic content (Figure 2b, Table 1). Further study of carbon density in Tasmanian saltmarshes in different estuaries and using replicated cores across species zones with statistical analysis could confirm these apparent trends.

The percentage carbon as shown by our results was also consistently lower than marshes studied elsewhere, ranging between 0.17% and 7.16%, likely influenced by grain size differences (Figure 2a). In marshes of the eastern USA, carbon percentages were found to range between 5.95% and 10.81% depending on marsh type [45], and in marshes of NSW, Australia carbon percentages were found to range between 3.7–5.9% for *J. kraussii* and 2.1–4.8% for *S. quinqueflora* [46]. This study, as indicated from one core per site, found lower carbon percentage ranges for the same species studied in NSW, of 0.17–7.16% for *J. kraussii* and 0.17–2.44% for *S. quinqueflora* (Figure 3).

It is likely that the overall lower levels of carbon found in the Rubicon estuary are due to higher net accretion rates (Table 1), dominated by inorganic sediment derived from the catchment. Net accretion rates under *J. kraussi* saltmarsh in NSW were found to be 1.74 ± 0.13 mm a^−1^, and under *Sarcocornia* 0.78 ± 0.18 mm a^−1^ [18], far lower than rates found in the Rubicon (Table 1). Over 35% of the Rubicon catchment area is used for agriculture, and a further 25% is in plantation forestry, with estimates of 0.3–5.3 Mg ha^−1^ a^−1^ of soil erosion from cropped and cultivated land [26,27]. Catchments in Tasmania have been relatively recently converted from native vegetation to productive land uses compared with many other locations in the world. Net accretion rates under *Spartina* in the adjacent Tamar estuary since its introduction have been 8.7–52.4 mm a^−1^ [11], indicating these higher regional trends. Carbon density results from this study are similar to saltmarsh research elsewhere, where high levels of inorganic sediments reduce organic matter accumulation [7,11,47].

However, the carbon accumulation rates as shown by core results from each of the sites within the Rubicon estuary indicated equivalence to global rates. Sites 2 and 3 showed carbon accumulation rates higher than the global average of 0.021 g cm^−3^ a^−1^ (including mangroves and saltmarshes) [7], while site 1.2 was similar in accreting 0.022 g cm^−3^ a^−1^ (Table 1).

Belowground carbon stocks of the Rubicon estuary saltmarsh (Table 2) were indicated to be low in comparison to all nine fluvial and marine saltmarsh sites analysed across NSW (29–37° S) [5], the mean carbon stock of saltmarshes globally [3], and results of carbon stocks across mainland Australia of average 78 (range 9–604) Mg OC ha^−1^ using the comparative measurement of the top 30 cm [17]. The Rubicon carbon evaluation of 49–55 Mg C ha^−1^ (Table 2) is most similar to results from sites in South Australia [17]. These carbon sequestration stores are of useful financial value in contribution to greenhouse mitigation according to recent auctions held by Australia’s Clean Energy Regulator [17].

This study indicates that the introduced *Spartina*, owing to its extent over 85% of the 265 ha of Rubicon saltmarsh area is likely contributing the majority of the sediment carbon store, estimated to be 12,543 Mg C (Table 2). Extensive coring of *Spartina* marshes in the adjacent Tamar estuary [11] showed *Spartina* to have accumulated >50 cm of sediment since its introduction last century in established areas and with mudflats beneath these deposits, suggesting that the 30 cm sediment depths analysed in the Rubicon are likely accumulated under *Spartina*. The finer grain sizes of sediment that *Spartina* has accumulated ([11], Figure 2a) would be an important influence.

Carbon accumulation rates and palaeo-environmental information for individual peatlands are often inferred from analyses conducted on a single core [48]. Analysis of 323 cores from 66 saltmarsh locations (several with up to three studies) across mainland Australia [17] included nine locations with just one core contributing to analysis of carbon sequestration trends. This Rubicon study contributes information from two cores from native marsh and two from *Spartina* marsh, however, the results could be confirmed by more extensive sampling, combined with statistical analysis. Studies on carbon storage from marshes in Victoria used three cores at each location [17], or four from each vegetation zone [5], and within-site differences in carbon accumulation and the complex responses of proxy indicators to both allogenic and autogenic changes [48] are improved by such practice.

Further work could also include pollen analysis of more cores, to confirm that the vegetation contributing to 30 cm depth of carbon accumulation, or 100 cm as analysed in other studies, is derived by the species presently on the surface. This study showed that this was the case for one native marsh site (Figure 4b), though the pollen of that species is generally poorly preserved so has reduced representation relative to other pollen. Pollen analysis of sediment cores is a low cost but time-consuming technique, where one diagram can require some months of careful laboratory analyses [49], however, this study confirms its potential value in confirming the sources of historic blue carbon stores. Dating of significant layers in the analysed levels of sediment [41] could also show the timeframe.

## 5. Conclusions

After its deliberate introduction last century, *Spartina* is now viewed in Australia as a problem invasive species [50,51] owing to its impacts on aquaculture, and threats to the ecological integrity of estuarine wetlands of international importance [11,30]. As a result, control and eradication strategies have been undertaken [30,42], such as limiting further spread onto mudflats that are important wader habitats. This study indicates that *Spartina* may have a previously unrealized value as a useful carbon sink, which could influence future management attitudes particularly towards the two largest infestations of this species in Australia of the Rubicon and Tamar estuaries, both in northern Tasmania. Largescale removal of *Spartina* has been shown in the Tamar to likely result in sediment erosion [11], and this study indicating its value as a carbon sink adds to caution in considering its large-scale removal from shorelines where it is now well-established. Future research could confirm the effects of invasive *Spartina* on increasing saltmarsh carbon sequestration in Tasmania.

Blue carbon of wetland sediment is of high value to greenhouse mitigation [17], and this study contributes saltmarsh sediment carbon information from the most southerly latitude reported to date. Overall, this study shows that the contribution of high latitude saltmarshes to carbon sequestration is of value, and the focus of most studies on lower latitude saltmarshes could be beneficially expanded to better understand trends.

## Figures and Tables

**Table 1:** Results from organic matter, bulk density, sediment carbon and carbon density determinations in the top 0.3 m of the cores from Rubicon estuary sites (mean ± SE).

Property	Site 1.1	Site 1.2	Site 2	Site 3
Vegetation type	*J. kraussii*	*S. quinqueflora*	*Spartina*	*Spartina*
Organic matter (%)	1.2 ± 0.2	1 ± 0.13	1.7 ± 0.2	1.8 ± 0.1
Bulk density (g/cm^3^)	1.47 ± 0.17	1.19 ± 0.11	0.68 ± 0.08	0.8 ± 0.16
Sediment carbon (%)	1.37 ± 0.63	0.78 ± 0.24	1.95 ± 0.39	2.6 ± 0.17
Carbon density (g/cm^3^)	0.023 ± 0.006	0.010 ± 0.002	0.017 ± 0.001	0.020 ± 0.002
Vertical accretion (mm/year)	0.42	18	20	30
Carbon accretion (g/cm^2^/year)	0.015	0.022	0.034	0.055

**Table 2:** Below ground carbon stocks (upper 30 cm) in the different saltmarsh species zones of the Rubicon estuary.

Saltmarsh Species Zone	Area (ha) [36,37]	Carbon Store (Mg C)	Carbon Stock (Mg C ha^−1^)
*Native saltmarsh*	39	1930	49.5
*Spartina*	226	12,543	55.5
